# Serum α-1 Antitrypsin (AAT) antagonizes intrinsic apoptosis induction in neutrophils from patients with systemic inflammatory response syndrome

**DOI:** 10.1371/journal.pone.0177450

**Published:** 2017-05-11

**Authors:** Theresia Sarabhai, Christoph Peter, Anne-Kathrin Bär, Joachim Windolf, Borna Relja, Sebastian Wesselborg, Thorsten Wahlers, Adnana Paunel-Görgülü

**Affiliations:** 1 Department of Trauma and Hand Surgery, University Hospital Düsseldorf, Düsseldorf, Germany; 2 Institute for Molecular Medicine I, Heinrich-Heine University Düsseldorf, Düsseldorf, Germany; 3 Department of Cardiothoracic Surgery, Heart Center of the University of Cologne, Cologne, Germany; 4 Department of Trauma, Hand and Reconstructive Surgery, University Hospital Frankfurt, Goethe University, Frankfurt, Germany; Hospital for Sick Children, CANADA

## Abstract

Excessive neutrophil activation accompanied by delayed apoptotic cell death in inflammatory conditions causes progressive damage of cells and tissues, leading to life-threatening multiple organ dysfunction syndrome. Previous work suggested that circulating serum factors during inflammation are critically involved in the suppression of neutrophil cell death although the identity of these antiapoptotic mediators remained elusive. In this study, we identified the acute phase protein α-1 Antitrypsin (AAT) as a potent suppressor of staurosporine (STS)-induced apoptosis in human neutrophils through a mechanism implicating caspases-independent pathways. We show here that serum levels of AAT, potentially in part released by stimulated neutrophils, are markedly elevated in major trauma patients suffering from systemic inflammatory response syndrome (SIRS). Notably, AAT depletion from serum increased sensitivity of human neutrophils for STS-induced cell death. In fact, AAT was demonstrated to confer intrinsic apoptosis resistance by preventing PKC/Akt inactivation and subsequent proteasomal degradation of antiapoptotic Mcl-1 protein in response to STS treatment. Neither MAP kinase ERK1/2 nor caspases were found to be involved in AAT-triggered antiapoptotic pathways in neutrophils. In summary, these results establish a novel pivotal role of circulating AAT in mediating survival by antagonizing the proapoptotic action of the PKC inhibitor STS and should be considered for AAT augmentation therapies in future.

## Introduction

Trauma is one of the major causes of mortality and morbidity in people under the age of 50 and care of patients with acute trauma causes costs of estimated 27 billion dollars per year in the USA alone [1]. Secondary complications such as acute respiratory distress syndrome, multiple organ failure and sepsis remain a significant cause of death in hospitalised trauma patients. It has been recently suggested that deregulated immune reactions are implicated in the development of such posttraumatic complication and that alterations in neutrophil function seem to play a decisive role.

Neutrophils or polymorphonuclear leukocytes are the most abundant white blood cells in human circulation. They are essential immune cells which determine the host’s resistance against bacterial and fungal pathogens and also participate in the development of the inflammatory reaction. Neutrophils have the shortest lifespan among leukocytes because neutrophil survival is limited by an intrinsic, constitutive apoptotic pathway [2].

The inflammatory response, such as after major trauma, can be characterized by increased expression of inflammatory cytokines, acute phase proteins and complement that result in the prolongation of neutrophil lifespan, cell activation and sequestration. Once activated, they rapidly migrate toward the injured site where they exert defensive functions, such as degranulation, release of reactive oxygen species, neutrophil extracellular traps and proteases, and pathogen elimination [3, 4]. Neutrophil activity is potentiated by host-derived inflammatory mediators such as IL-8 and GM-CSF which might prolong neutrophil survival by activation of intracellular survival pathways and inhibition of apoptosis [5, 6]. However, increased neutrophil number and activity due to dysregulated apoptosis can lead to bystander tissue damage and might contribute to the pathogenesis of inflammatory diseases, such as systemic inflammatory response syndrome (SIRS), sepsis and acute respiratory distress syndrome [7–9]. In addition, neutrophils were demonstrated to directly interact with immune cells, including T and B cells, NK cells and DCs which emphasizes their capacity to contribute to the modulation of adaptive immunity and probably immunosuppression [10]. Therefore, the apoptotic cell death is critical for termination of the inflammatory response and diminution of neutrophil activity, thus preventing injury of host tissues.

Probably one of the most investigated proteins related to neutrophil apoptosis is the antiapoptotic factor myeloid cell leukemia 1 (Mcl-1). Mcl-1 accumulation protects against formation of the Bak/Bax heterodimer on the external mitochondrial membrane and subsequent release of cytochrome c, SMAC/Diablo, AIF from the mitochondrial intermembrane space. Thus, Mcl-1 is critical for the regulation of mitochondrial transmembrane potential and suppression of the intrinsic apoptosis pathway [11]. Delayed neutrophil apoptosis found in a wide variety of inflammatory diseases often correlates with the severity / outcome of the disease as well as with intracellular Mcl-1 protein levels [12, 13]. In this regard, activation of pI3K/Akt, ERK1/2 and PKC, among others, during acute inflammation have already been demonstrated [6, 14, 15]. Directly relevant to apoptosis, Akt-mediated phosphorylation inactivates caspase-9 and prevents Bax association with mitochondria. Inactivation of another Akt target, GSK3-ß, prevents phosphorylation, ubiquitination, and proteasomal degradation of Mcl-1 [16]. Additionally, activation of PKC was found to be critical for neutrophil function in inflammation and inhibition of PKC activity has been proposed as potential antiinflammatory therapy [17].

Delayed apoptosis of neutrophils from major trauma patients with SIRS has previously been reported by our group to be associated with increased expression of antiapoptotic Mcl-1 and apoptosis resistance to the PKC inhibitor staurosporine (STS) [18]. Patients’ serum completely prevented STS-mediated inactivation of Akt kinase and subsequent Mcl-1 degradation in neutrophils from healthy volunteers [16]. In the current study, we aimed to identify serum factors conferring resistance to the PKC inhibitor STS during systemic inflammation by performing affinity chromatography and mass spectrometric analyses. Further on, the mechanism of action should be validated to assess the therapeutic potential of the newly identified factor(s) in critical care.

## Materials and methods

### Material

α-1 Antitrypsin isolated from human plasma by chromatography was purchased from Sigma-Aldrich (A9024).

### Study population

This study was approved by the Ethics Committee of the University of Düsseldorf (#3412), Germany and parts of the study were approved by the Ethics Committee of the Goethe University Frankfurt, Germany (#312/10). The investigations were carried out according to the principles outlined by the Declaration of Helsinki. Written informed consent was obtained from all participants or their legal representatives. Patients with blunt or penetrating multiple injuries who were admitted to our Level I Trauma Center with an Injury Severity Score (ISS) ≥ 16, intensive care unit (ICU) stay > 3 days and aged 18 years or older were enrolled in this study. Blood was collected from patients at days 1–2, days 5–6 and days 10–11 after admission. Sera were harvested by centrifugation and stored at -80°C until further processing.

Human neutrophils were isolated from the whole blood of healthy volunteers or buffy coats obtained by employees at the blood bank of the university. Inclusion criteria were: age >18 and absence of disease or physical discomfort. Exclusion criteria were: immunological disorders and immunosuppressive medication, chronic diseases, and *in vitro* STS resistance of freshly isolated, unstimulated neutrophils.

### Isolation of human neutrophils

Human neutrophils were separated from whole blood or buffy coats by discontinuous density-gradient centrifugation on Percoll (Biochrom) as previously described [18]. After hypotonic lysis to remove contaminating erythrocytes, cells were washed with in PBS. Purity and viability were routinely > 95% as assessed by forward and side scatter characteristics of FACSCalibur (BD Biosciences) and trypan blue exclusion, respectively.

### Cell culture

HL-60 cells were grown in RPMI 1640 (Biochrom) supplemented with 10% heat-inactivated FCS, 100 U/ml penicillin, 100 μg/ml streptomycin in a 5% CO_2_ humidified atmosphere at 37°C. Cells (2 × 10^6^) were cultured in the presence of all-trans-retinoic-acid (ATRA; Sigma) in a final concentration of 1 μM for 5 days. Cell differentiation was assessed by apoptosis rate quantification by flow cytometry (FACSCalibur, BD).

In some experiments, freshly isolated human neutrophils, the Raji line of lymphoblast-like cells, HL-60, ATRA-differentiated HL-60 cells, Jurkat and THP1 cell lines were cultured in RPMI 1640 medium supplemented with 100 U/ml penicillin, 100 μg/ml streptomycin and 1% or 3% of serum, respectively.

### Dialysis of human serum

In order to sub-classify serum proteins according to their particle size, pooled patients’ serum was given into a semipermeable membrane system (2 kDa, 10 kDa, 20 kDa Slide-a-Lyzer, Dialysis Cassetts, Thermo Scientific). Incubated at 4°C, the membranes were allowed to float in 1 × PBS overnight. Additionally, to exclude proteins with a molecular weight below 50 kDa, dialysis with a Float-A-Lyzer G2 dialysis device (molecular weight cut off 50 kDa; Spectrum Labs) was performed. After dialysis, the remaining sera were stored at -80°C until further processing or usage in cell culture.

### Chromatographic depletion of albumin from human serum

All chromatography steps were carried out on an Äktapurifier Workstation from GE with prepacked columns from GE Healthcare. For removal of albumin serum from severely injured patients was passed manually over a Cibacron Blue affinity column (5 ml HiTrap Blue). Albumin and the bound nonalbumin proteins were eluted with 2 M NaCl in 20 mM sodium phosphate (pH 7.2). The albumin-depleted serum was collected and separately stored at -80°C until further processing.

Analytical size exclusion chromatography was achieved by fractionating albumin-depleted serum (150 μg/100 μl) on a Superose 6 10/300 GL gel filtration column. After chromatographic processing the serum fractions were stored at -80°C until further processing.

### Depletion of Ig from human serum

To reduce the Ig content, pooled patients’ serum was mixed with Protein G Plus/Protein A Agarose beads (Calbiochem, Darmstadt, Germany) in the ratio 10:1 and incubated for 1 h with gentle shaking at 4°C. Then, samples were spun down and supernatants were transferred into a fresh tube. Ig depletion has been performed for a total of eleven times. Supernatants were stored at -80°C and precipitated beads were stored at 4°C until further processing.

### AAT immunoprecipitation from human serum

For immunodepletion of AAT from pooled serum from polytraumatized patients, anti-α-1 antitrypsin antibody (Abcam, ab207303) was precoupled to Protein G Plus/Protein A Agarose beads (Calbiochem, Darmstadt, Germany) by incubation at 4°C overnight. Then, patient serum was diluted to a final concentration of 3 mg protein/ml and serum samples were incubated with antibody-loaded beads and further incubated with rotation at 4°C overnight. After incubation, beads were pelleted by centrifugation and AAT-depleted supernatants were collected and stored at -80°C under further processing.

### Mass spectrometry and identification of proteins

Proteomic analyses were performed by the Molecular Proteomics Laboratory at the Biologisch-Medizinisches Forschungszentrum (BMFZ) of Heinrich-Heine University Düsseldorf. Liquid chromatography electrospray ionization mass spectrometry was used for the identification of proteins from human serum sample. 5 μg of the sample was separated in a 4 to 12% bis-tris polyacrylamide gel (Life Technologies, Darmstadt, Germany). After a MS-compatible silver staining, the protein containing lane was cut out, destained, reduced and alkylated. The trypsin digestion was done overnight at 37°C. The peptides were extracted from the lane with a solution of 0.1% trifluoroacetic acid and acetonitrile (1:1 (v/v)). For the LC-MS/MS analysis the solvent was removed and 400 ng peptides were subjected to liquid chromatography.

An UltiMate 3000 RSCLnano System (Dionex/Thermo Fisher Scientific, Idstein, Germany) was used to separate the peptides. After the injection of the sample the peptides were pre-concentrated on a C18-pepmap column (Acclaim PepMap 100; 2 cm length, 75 μm inner diameter, 3 μm particle size, 100 Å pore size; Dionex/Thermo Fisher Scientific, Idstein, Germany) with a flow rate of 6 μL/min. After 10 min the separation was started on an analytical C18 column (Acclaim PepMap 100-RSLC; 25 cm length, 75 μm inner diameter, 2 μm particle size, 100 Å pore size; Dionex/Thermo Fisher Scientific, Idstein, Germany). A 120 min gradient was used from 4 to 40% solvent B (A: 0.1% (v/v) formic acid in water B: 0.1% (v/v) formic acid, 84% (v/v) acetonitrile in water) with a flow rate of 300 nL/min.

Mass spectrometry was performed on an Orbitrap Elite high resolution instrument (Thermo Fisher Scientific, Bremen, Germany). The peptides were directly eluted by nano electrospray ionization (voltage: 1.4 kV) using a nanosource interface. The MS instrument was operated in the positive mode with a mass range 350–1700 m/z and a resolution up to 60,000. The target value for the automatic gain control was 1,000,000 with the maximum fill time of 200 ms. The 20 most intense doubly and triply charged peptide ions (minimal signal intensity of 500) were isolated, transferred to the linear ion trap (LTQ) part of the instrument, and fragmented by collision-induced dissociation (mass range of 200–2000 m/z at a resolution of 5,400). The fragments of the peptides were analyzed with a maximal fill time of 300 ms and automatic gain control target value of 10,000, already fragmented peptides were automatically excluded for the fragmentation for 45 s.

The generated raw-Files were analyzed using Proteome Discoverer (version 1.4.1.14, Thermo Fischer, Dreieich, Germany) connected to a Mascot server (version 2.4, Matrix sciences, London, UK) with default parameters for spectrum selection. The SwissProt database (version SwissProt_2014_06) was used for the peptide identification with the following parameters: mass tolerance of 10 ppm or 0.4 Da, trypsin specific cleavage with a maximum of one missed cleavage site, the oxidation of methionine as dynamic modification and carbamidomethyl as static modification. The false discovery rate (FDR) on peptide and protein level was below 1%. Known contaminations were removed from the protein list. A minimum of 2 unique peptides were required for identification as well as quantification. All proteins were quantified only based on unique peptides.

### Western blotting

Isolated neutrophils were resuspended in RIPA buffer (50 mM Tris pH 8.0, 150 mM NaCl, 1% Nonidet P-40, 0.5% sodium deoxycholate, 0.1% SDS) supplemented with protease and phosphatase inhibitor mixture (both Cell Signaling) followed by cell sonication. Samples were separated on SDS-PAGE and transferred to nitrocellulose membranes (Peqlab, 0.2 μm). The following antibodies were used: mouse monoclonal anti-human Mcl-1 (BD Pharmingen, 559027), rabbit polyclonal anti-human Mcl-1 (phospho S159; Abcam, ab111574), rabbit polyclonal Phospho-Erk1/2 (Thr202/Tyr204, Cell Signaling, 9101), rabbit polyclonal Erk 1/2 (Cell Signaling, 9102), rabbit monoclonal Akt (Cell Signaling, 4691), rabbit polyclonal Phospho-Akt (Ser473) (Cell Signaling, 9271), monoclonal mouse anti-human Serpin A1/α1-Antitrypsin (R&D Systems, MAB1268), mouse monoclonal GAPDH (Novus Biologicals, NBP2-27103). The membranes were further incubated with polyclonal goat anti-mouse immunoglobulins/HRP (Dako, P0447) or polyclonal goat anti-rabbit immunoglobulins/HRP (Dako, P0448), respectively. For detection of human immunoglobulin, a HRP-conjugated goat-anti human IgG + IgA + IgM (H+L) antibody (Biozol, MBS539207-2) has been used. Bands were visualized using Pierce ECL Western Blotting Substrate (Thermo Fisher Scientific, 32209) and UptiLight HRP Blot Chemiluminescent ECL Substrate (Uptima, UP99619A).

### Analysis of AAT gene expression by Real time PCR

Total RNA from neutrophils was extracted using TRI Reagent (Sigma) according to the manufacturer’s instructions. Contaminating DNA was removed by digestion with DNase (DNA-*free* DNA removal kit, Ambion). 200 ng RNA were reverse transcribed to cDNA using oligo(dT)_15_ primer, random primer, and Omniscript Reverse Transcriptase (Qiagen). Gene-specific primer pairs for human AAT [19] and 18S [20] have been used. Relative gene expression levels were determined using SYBR Green (Applied Biosystems) incorporation following the manufacturer’s recommended protocol with the following thermal cycling conditions: 95°C, 10 min (1 cycle); 95°C, 15 sec, 60°C, 60 sec (40 cycles); 4°C hold. All samples were run in triplicates (ABI Prism 7300, Applied Biosystems). Expression of the AAT gene was normalized to the 18S RNA gene. Fold expression was calculated using the 2^-ΔΔC^_T_ method [21].

### Quantification of AAT in patients’ serum

Serum levels of human AAT were determined by a commercially available Elisa kit (Human Alpha 1-Antitrypsin Elisa Kit, Genway, GWB-5428A0) according to manufacturer’s protocol.

### Quantification of caspase activity

Neutrophils cultured in the presence of AAT-reduced patient serum and further treated with STS were harvest by centrifugation and pellets were stored at -80°C for further investigation. Caspase activities were measured by using Caspase-Glo^®^ 9 Assay and Caspase-Glo^®^ 3/7 Assay (both Promega) according to the instructions of the manufacturer with some modifications. In brief, cells were suspended in ice-cold PBS and sonicated. 100 μl of cell lysates containing 5 μg of protein were used for the determination of caspase activity. Luminescence was measured using VictorX3 plate reader (Perkin Elmer).

### Determination of neutrophil apoptosis

Neutrophil apoptosis was assessed as previously described [18]. In brief, neutrophils were suspended in hypotonic lysis solution (0.1% sodium citrate, 0.1% TritonX-100) containing 50 μg/ml propidium iodide and incubated in the dark at 4°C for 2 h before analysis by flow cytometry (FACSCalibur, BD). The fluorescence of propidium iodide bound to DNA was measured using Cell Quest software (BD) for cell acquisition and data analysis. The percentage of hypodiploid DNA (sub-G1) corresponding to fragmented DNA characteristic for apoptotic cells was calculated.

### Statistical analyses

Data were analyzed with GraphPad Prism 5 sofware. Data are presented as means with standard error of the mean (SEM). Some data are presented as box plots representing the median (heavy line in boxes) and the 25^th^ and 75^th^ percentiles. Whiskers indicate the minimum and maximum values, respectively. To evaluate differences between nonparametric data, the Kruskal-Wallis test with Dunns post hoc test was performed. Normally distributed unpaired data of multiple groups were analyzed with one-way ANOVA and Newman keuls post-hoc test. Unpaired data of two groups were analyzed using the t-test. P-value less that 0.05 was considered as statistically significant.

## Results

### Serum from severely injured patients confers resistance to STS in different cell lines

**Fig 1:**
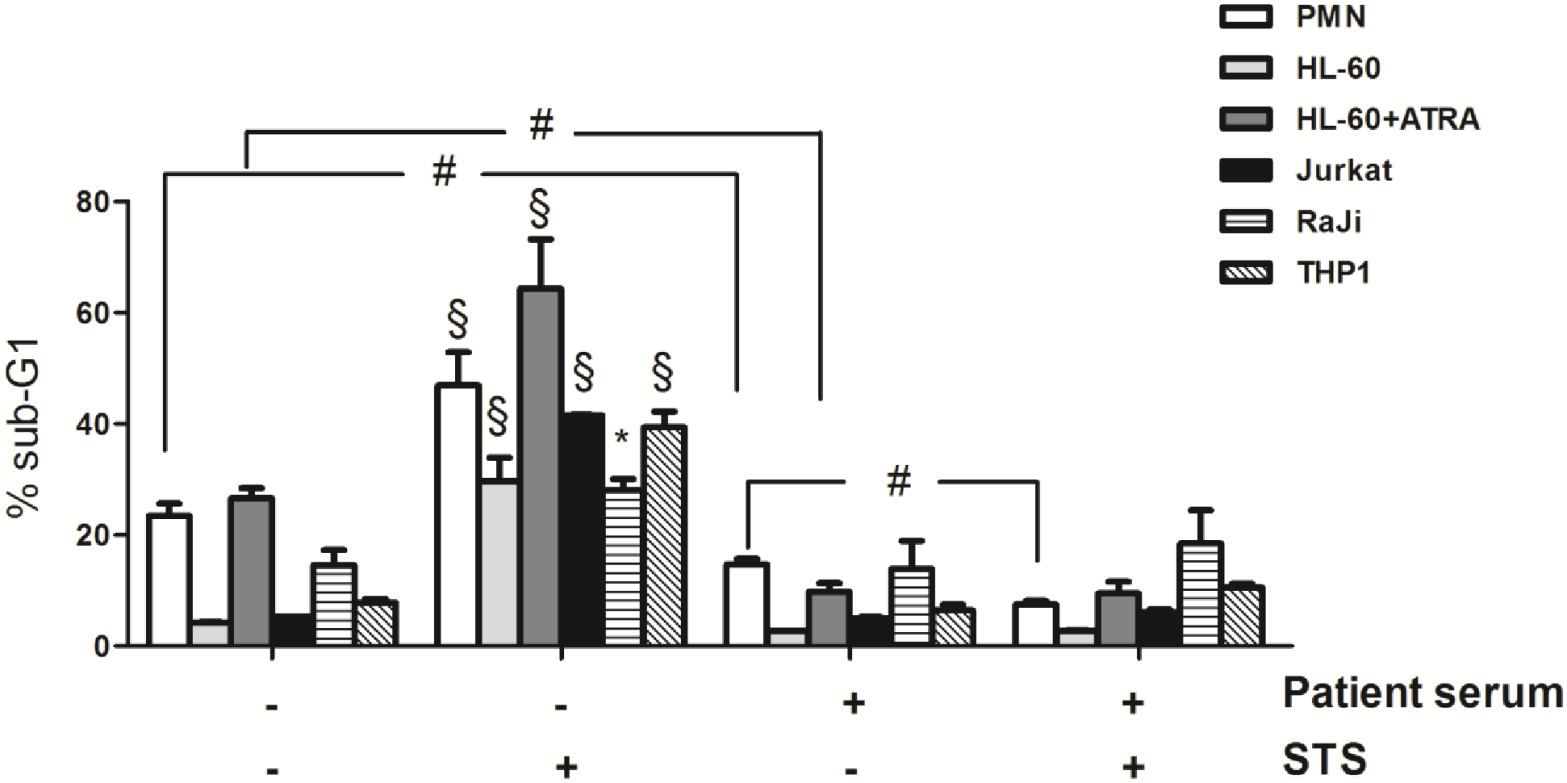
Serum from SIRS patients confers STS resistance in different cell types. Cells (2 × 10^6^/ml) were cultured in the presence or absence of 1% pooled patients serum (days 1–3 after admission) and further treated with 0.2 μM STS for 18 h. Then, apoptosis rate was quantified by propidium iodide staining of fragmented DNA and flow cytometry. Percentage of control neutrophils (n = 10), HL-60 cells (n = 3), ATRA-differentiated HL-60 cells (n = 6), RaJi, Jurkat, and THP-1 cells (each n = 3) with fragmented DNA (% sub-G1) is depicted. Data are presented as means ± SEM. N represents the number of replications for each experiment. *p<0.05, §p<0.001 vs. untreated control cells (w/o serum, w/o STS); #p<0.05 (one-way ANOVA with Newman keuls post-hoc test).

In our previous studies we have already demonstrated that neutrophils isolated from severely injured patients display strong intrinsic apoptosis resistance when cultured in the presence of the potent PKC inhibitor STS. Notably, patient sera was found to confer STS resistance in neutrophils from healthy volunteers to a similar degree as found in patients cells [16, 18]. To elaborate if serum-mediated STS resistance is cell type restricted, we first tested STS resistance in several myeloid and lymphoid lineages of blood cells. As depicted in Fig 1, all cell lines tested, including promyelocytic HL-60 cells, ATRA-differentiated neutrophil-like HL-60, lymphocytic Jurkat cells, Raji lymphoma cell line and THP-1 monocytes showed strong resistance to STS-induced apoptosis when cultured in the presence of pooled serum from severely injured patients to a similar degree as found in neutrophils isolated from healthy volunteers. Thus, serum-mediated apoptosis resistance to STS affects many cell types arguing for similar regulatory mechanisms. Serum supplementation alone significantly reduced apoptotic cell death in primary neutrophils and ATRA-differentiated HL-60 cells. Of note, treatment of serum-primed cells with STS decreased apoptotic cell death in neutrophils only, confirming the antiapoptotic effect of STS found in these cells [16]. Below, only primary neutrophils were used for further analyses.

### Serum low molecular molecules, albumin and immunoglobulins are not involved in cellular pathways conferring STS resistance

**Fig 2:**
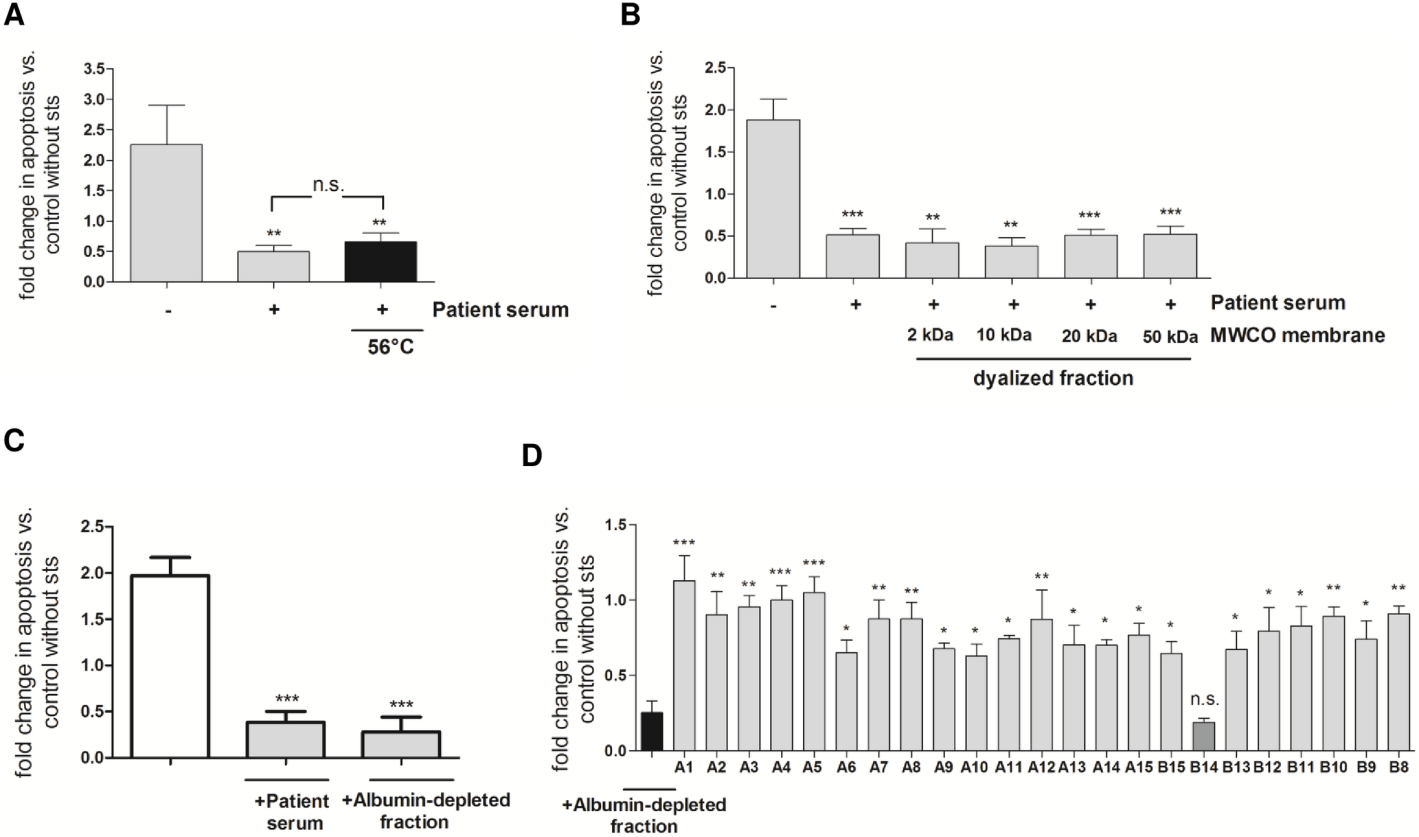
STS resistance is mediated by high molecular, albumin-independent serum ingredients. **A.** Pooled serum (days 1–3 after admission) was heat inactivated for 20 min at 56°C. Then, neutrophils from healthy donors (2 × 10^6^/ml) were cultured in medium supplemented with 1% serum and treated with 0.2 μM STS for 18 h. Cells cultured in the absence of patient serum were used as control. Fold change in apoptosis compared to cells without STS treatment is depicted. Data are expressed as means ± SEM of ten independent experiments. **p<0.01 compared to control sample w/o patient serum (one-way ANOVA with Newman keuls post-hoc test). **B.** Pooled serum was dialyzed using different membrane pore sizes (2–50 MWCO). Neutrophils were cultured in medium supplemented with dialyzed serum fractions or original patient serum (each 1%) and treated with STS as already mentioned above. Fold change in apoptosis vs. control cells without STS treatment is depicted. Results (means ± SEM) from three independent experiments are depicted. **p<0.01, ***p<0.001 compared to control sample w/o patient serum (one-way ANOVA with Newman keuls post-hoc test). **C.** Heat-inactivated serum from patients was reduced in albumin by affinity chromatography using a HiTrap Blue HP column. Then, neutrophils were cultured in medium supplemented with patients’ serum or the albumin-depleted fraction (3%) respectively and further treated with STS (0.2 μM) for 18 h. Relative apoptosis vs. control cells without STS treatment is depicted. Data are presented as means ± SEM of four independent experiments. ***p<0.001 compared to control sample w/o patients serum (one-way ANOVA with Newman keuls post-hoc test). **D.** Heat-inactivated, albumin-depleted serum fraction (150 μg/100 μl) was injected to the HPLC system fitted with a Superose 6 10/300 GL gel filtration column. Neutrophils from healthy donors were cultured in medium supplemented with the fractions (A8-A15; B8-B15, each 10%) and treated with STS. Fold change in apoptosis vs. control cells without STS treatment is depicted. Data are presented as means ± SEM of three independent experiments. *p<0.05, **p<0.01, ***p<0.001 as compared to the albumin-depleted fraction (black bar); n.s. = not significant (one-way ANOVA with Newman keuls post-hoc test).

Heat treatment of serum for 20 min at 56°C did not diminish STS resistance of primary human neutrophils arguing against a direct role of complement factors (Fig 2A). To identify factor(s) which trigger intrinsic apoptosis resistance, serum proteins of low molecular weight were removed by performing dialysis using dialysis membranes with different molecular weight cut offs (ranging from 2 kDa to 50 kDa). The exclusion of molecules below 50 kDa did not influence STS resistance in human neutrophils suggesting that the molecular weight of molecules involved in apoptosis resistance is greater than 50 kDa (Fig 2B).

**Fig 3:**
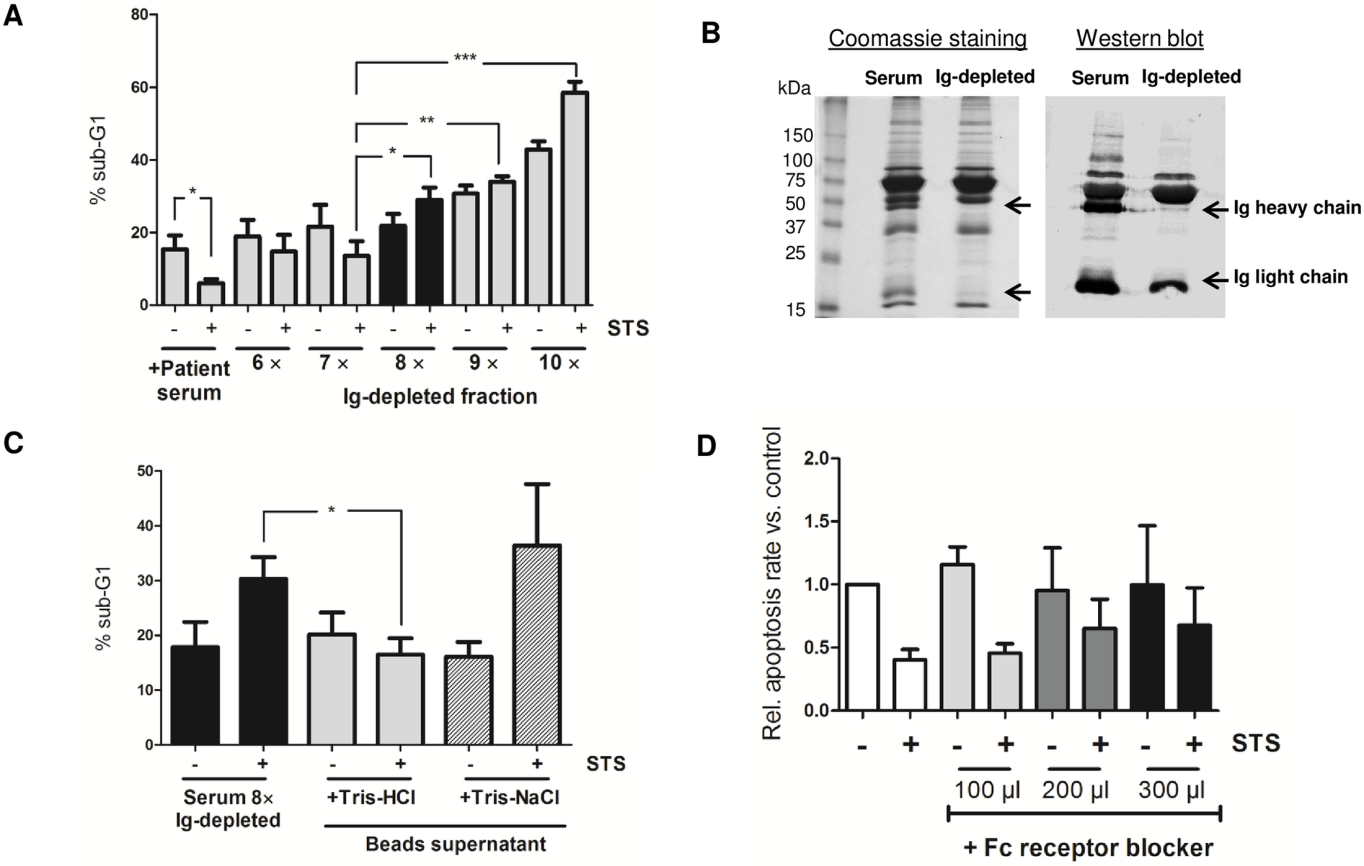
STS resistance does not depend on immunoglobulins or Fc receptor-triggered pathways. **A.** Pooled patient serum was subjected to serial depletion of immunoglobulins (Ig) using Protein G Plus/Protein A Agarose. Beads supernatant was collected after each depletion step. After 10 depletion steps, neutrophils from healthy volunteers (2 × 10^6^/ml) were cultured in the presence of the Ig-depleted fractions and tested for intrinsic apoptosis resistance. Cells were treated with STS (0.2 μM) for 18 h and apoptosis was determined by flow cytometry. Data are presented as means ± SEM of four to eight independent experiments. *p<0.05, **p<0.01, ***p<0.001 (t-test). **B.** Serum depleted for Ig for 8 times was subjected to SDS-PAGE and the gel was stained with Coomassie Brillant Blue (*left*). In addition, immunoblot analysis using Ig-specific antibody was performed (*right*). Arrows indicate the light and heavy chain of Ig. **C.** Beads used for the 8^th^ depletion step were washed with Tris-HCl (25 mM, pH 7.5) and Tris-NaCl (25 mM Tris-HCl pH 7.5, 1 M NaCl), respectively, and supernatants were collected. Neutrophils were cultured in the presence of Ig-depleted serum, Tris-eluted supernatant (each 1%) and STS (0.2 μM) as already described. The percentage of cells displaying fragmented DNA (% subG1) is depicted. Graphs show means ± SEM of four independent experiments. *p<0.05 (t-test). **D.** Neutrophils were cultured in the presence of three different concentrations of Fc receptor blocker, using a peptide based technology, and patient serum (1%) and further treated with STS (0.2 μM). Relative apoptosis rate vs. control sample without Fc receptor blocker and STS is displayed. Data (means ± SEM) from five independent experiments are presented. No significant differences in apoptosis could be found when compared to the STS-treated control without Fc blocker (t-test).

The plasma proteome consists of a large variety of proteins that are present in different concentrations. On a logarithmic scale, a range of 9 to 12 orders of magnitude can be expected for the abundance of different protein species in serum. Several high-abundance proteins, such as albumin, immunoglobulins (Ig), haptoglobin, transferrin, typically constitute 85–90% of total plasma proteins. These dominant proteins may mask the determination of low-abundance proteins that are of special interest. Detection of low-abundance proteins therefore requires the specific depletion of high-abundance proteins by different approaches, such as affinity chromatography. Therefore, pooled patient serum, which has been previously heat treated, was reduced for serum albumin by affinity chromatography using a HiTrap blue column. However, albumin depletion did not compromise neutrophil STS resistance (Fig 2C). Considering that the factors of interest are included in the albumin-depleted serum sample, this fraction was further subjected to Superose 6 column chromatography. Then, single fractions were tested in cell culture for their ability to induce STS resistance in human neutrophils from healthy volunteers (Fig 2D). Interestingly, only one fraction (B14) was found to protect neutrophils from STS-induced cell death. In parallel, serum was depleted for Ig using Protein G Plus/Protein A Agarose beads for a total of ten times (Fig 3A). Ig depletion was verified by SDS-PAGE, coomassie staining of the gel and immunodetection of human Ig light and heavy chain by western blot analysis (Fig 3B). To further explore if Ig-depletion influences STS resistance, human neutrophils were incubated in the presence of the Ig-depleted serum fractions and STS sensitivity was tested. As shown in Fig 3A, we found STS resistance in neutrophils to be significantly abolished after 8 times of Ig serum depletion suggesting that molecules responsible for intrinsic apoptosis resistance were withdrawn at this depletion step. To identify Ig-associated factors, which have been (co)immunoprecipitated, agarose beads were washed with Tris-HCl (25 mM, pH 7.5) or Tris-NaCl (25 mM Tris-HCl pH 7.5, 1 M NaCl) respectively and supernatants were added to neutrophils cultured in presence or absence of STS. We observed, that culturing of neutrophils in medium supplemented with Tris-HCl supernatant, but not Tris-NaCl, restored STS resistance (Fig 3C). This result strongly indicates that regulatory factors of neutrophil intrinsic apoptosis were eluted from the beads. To further exclude that serum-mediated effects on neutrophil apoptosis are linked to Ig receptors, we next blocked the Fc receptors on neutrophils before serum supplementation and addition of STS to the culture medium. Our results confirm that STS resistance is not mediated via Fc receptors and other molecules than Ig might be implicated (Fig 3D).

Having demonstrated that the factors of interest might be included in the B14 fraction as well as in the Tris-eluted supernatant, these fractions were further loaded onto a bis-tris gel before SDS-PAGE. Major protein bands were cutted out and analyzed by mass spectrometry. Beside several high abundant serum proteins, α-1 Antitrypsin (AAT) has been identified (S1 and S2 Tables).

### Human plasma-derived AAT abrogates the proapoptotic action of STS

**Fig 4:**
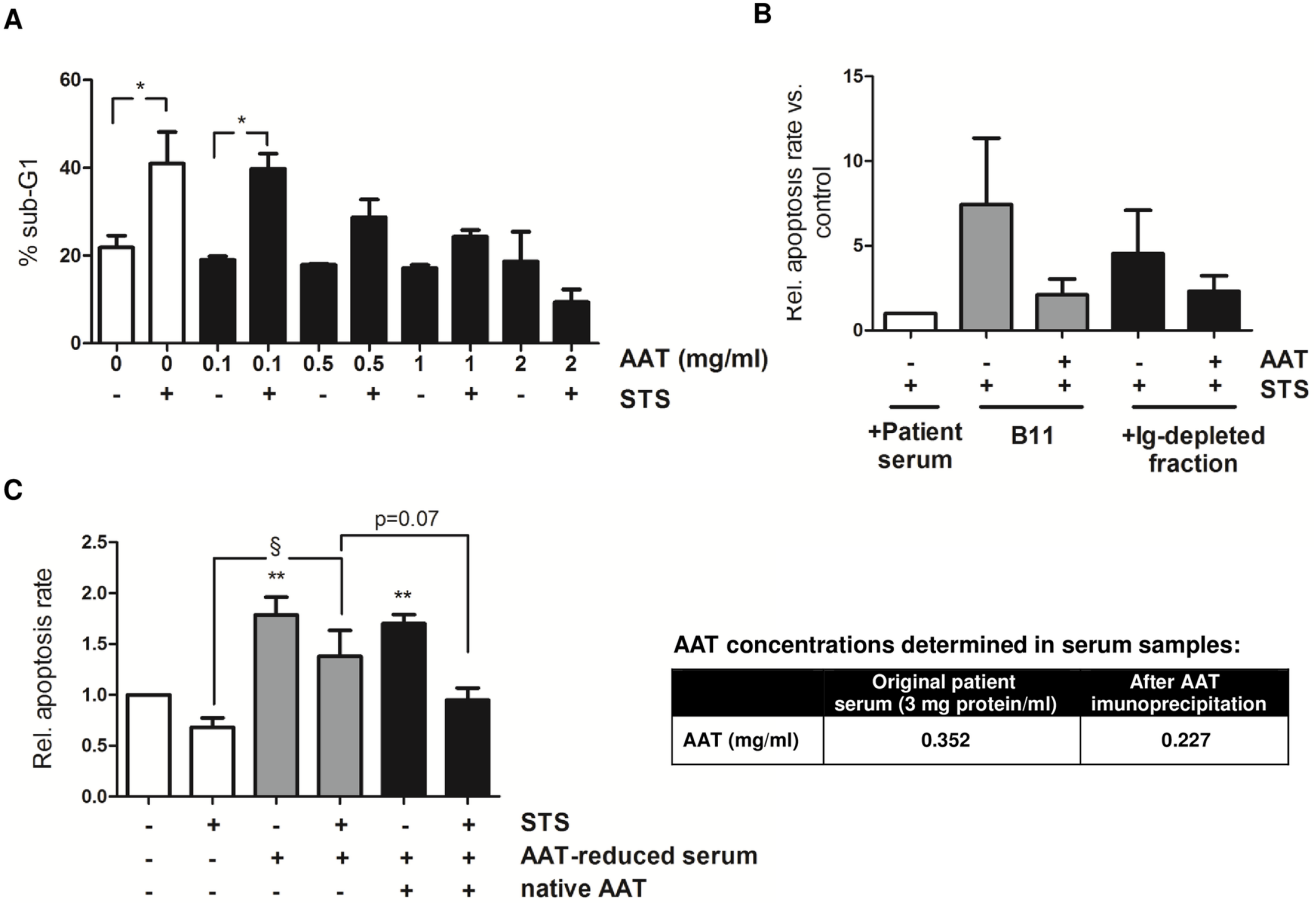
Serum AAT confers resistance to STS in human neutrophils. **A.** Neutrophils from healthy volunteers (2 × 10^6^/ml) were cultured in medium supplemented with different concentrations of plasma-derived human AAT (range 0–2 mg/ml) and treated with STS (0.2 μM). After 18 h, apoptosis was quantified by propidium iodide staining and FACS analysis. Percentage of apoptotic cells displaying fragmented DNA is depicted. Results are presented as mean ± SEM of three independent experiments. *p<0.05 (t-test). **B.** Neutrophils cultured in medium supplemented with the albumin-depleted fraction B11 and the serum fraction depleted for Ig for 9 times (3%), which have been shown to not confer resistance to STS, were additionally pre-incubated with 2 mg/ml plasma-derived AAT. After 1 h, STS (0.2 μM) was added and cells were further cultured overnight. Data of three independent experiments are expressed as fold changes vs. control cells cultured in the presence of patient serum. No significant differences could be found. **C.** Pooled patient serum was first diluted to a final concentration of 3 mg protein/ml. Then, serum was reduced for AAT by using an AAT-specific antibody and AAT decrease was further confirmed by Elisa. Empty beads were used as control. AAT concentrations are depicted in the table on the right. Patient serum, AAT-reduced serum (each 1%) with or without plasma-derived human AAT (native AAT; 2 mg/ml) were added to the culture medium of neutrophils (2 × 10^6^/ml). After treatment with STS (0.2 μM) for 18 h, apoptosis rate was quantified. Fold changes in apoptosis vs. control cells are displayed. Graphs show results of four independent experiments. **p<0.01 vs. untreated control; §p<0.05 (one-way ANOVA with Newman keuls post-hoc test).

To prove the modulatory role of serum AAT on neutrophil survival, freshly isolated neutrophils from healthy volunteers were cultured in medium supplemented with plasma-derived AAT (Sigma) and treated with STS. As depicted in Fig 4A, AAT alone did not markedly influence neutrophil spontaneous cell death, but significantly suppressed STS sensitivity in a concentration-dependent manner. To further validate that AAT supplementation confers STS resistance, we incubated cells in medium containing the albumin-depleted fraction B11 (Fig 2D) and the fraction depleted for Ig for nine times (Fig 3A), both having previously been shown to not protect against STS-induced cell death. As displayed in Fig 4B, AAT added to the culture medium markedly decreased cell sensitivity to proapoptotic STS. Contrary, culturing of cells in the presence of pooled patient serum with reduced AAT levels after immunoprecipitation increased STS-triggered apoptosis and spontaneous apoptosis, respectively. However, although AAT supplementation of AAT-reduced serum diminished STS-induced cell death in cultured neutrophils (Fig 4C), acceptable statistical significance was not achieved (p = 0.07).

### Neutrophil AAT expression after major trauma

**Fig 5:**
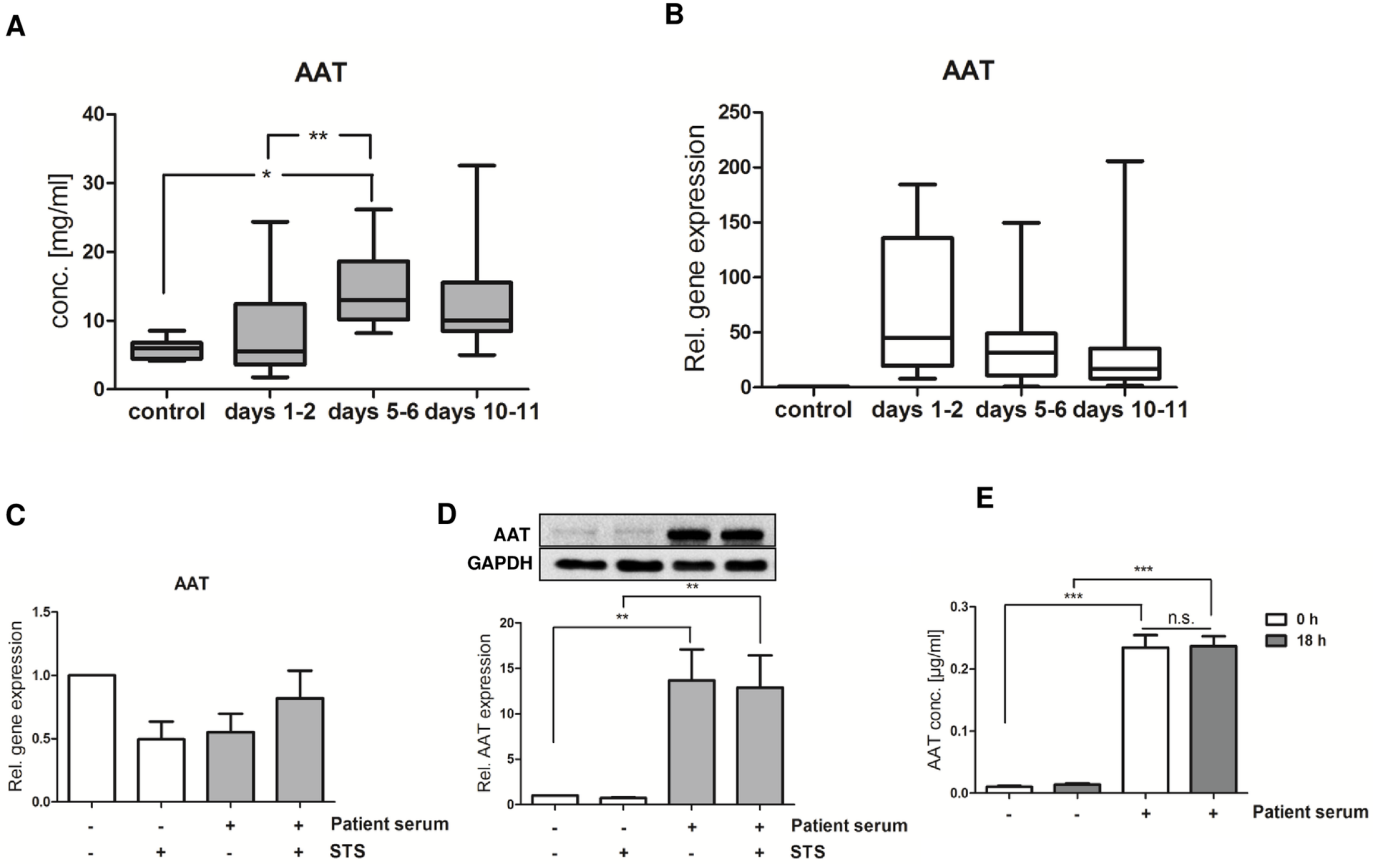
Serum from trauma patients induces intrinsic apoptosis resistance by up-regulating AAT expression in neutrophils. **A.** Levels of AAT were quantified in the serum isolated from healthy volunteers (n = 6) and major trauma patients (n = 13) at days 1–2, days 5–6 and days 10–11 after admission by Elisa. *p<0.05; **p<0.01 (Kruskal-Wallis test with Dunns post hoc test) **B.** Additionally, AAT gene expression levels were quantified in neutrophils isolated from healthy volunteers and trauma patients by Real time PCR. Gene expression was normalized to that of 18S RNA. Expression levels are reflected as fold-change of expression relative to control levels (healthy volunteers). No significant differences were found. **C.** Neutrophils from healthy donors (2.5 × 10^6^/ml) were cultured in medium containing FCS (1%) or pooled patient serum (1%) and treated with STS (0.2 μM). After 14 h, 3 μg/ml Brefeldin A was added to the cultures and cells were further cultured for additional 4 h. AAT gene expression was quantified by Real time PCR. Values were normalized to 18S RNA expression. Results of seven independent experiments are depicted. No statistical differences could be detected (one-way ANOVA with Newman keuls post-hoc test). **D.** AAT protein expression was analyzed in neutrophils after 18 h of culture by western blot analysis. Relative expression was quantified vs. GAPDH expression. One representative blot of five independent experiments is depicted. **p<0.01 (one-way ANOVA with Newman keuls post-hoc test). **E.** Neutrophils from healthy donors (2.5 × 10^6^/ml) were cultured in medium containing FCS (1%) or pooled patient serum (1%) overnight. AAT levels in culture supernatants were quantified by Elisa at 0 h as well as after 18 h. Results of three independent experiments are depicted. ***p<0.001; n.s. = not significant (one-way ANOVA with Newman keuls post-hoc test).

Previously, we found that neutrophils isolated from severely injured patients display an impaired intrinsic apoptosis pathway until at least day 10 after trauma [20]. We therefore addressed the question, whether STS resistance is correlated to AAT levels in patients’ serum. Interestingly, AAT serum levels were only slightly elevated during the first 48 hours after trauma and peaked at days 5–6. At days 10–11, AAT levels started to decline but remained above control levels (Fig 5A). Several years ago, human neutrophils themselves have been found to express AAT [22]. Indeed, in our cohort of severely injured patients AAT gene expression was markedly upregulated in neutrophils, but did not reach level of significance because of great interindividual variability (Fig 5B). Nevertheless, these data suggest that expression of AAT mRNA in neutrophils is widely accompanied by elevated AAT serum levels, and vice versa. On the basis of these data, we further speculated that neutrophils might represent a potential source for AAT after major trauma and that intrinsic AAT expression is linked to STS resistance. To prove this hypothesis, freshly isolated neutrophils from healthy volunteers were cultured in medium supplemented with patient serum or not and further treated with STS. We show here that serum from trauma patients did not alter AAT gene expression in cultured neutrophils (Fig 5C). Of note, a strong increase in intracellular AAT protein could be detected in serum-primed cells but not in control cells without intrinsic apoptosis resistance (Fig 5D). Apoptosis resistance and AAT upregulation do not seem to be based on AAT uptake from the extracellular space, as AAT concentrations in culture supernatants did not change over time (Fig 5E). However, our data also demonstrate that newly synthesized AAT is efficiently retained in the cells. Whether AAT expressed by neutrophils themselves, circulating AAT, or even both are mandatory for intrinsic apoptosis resistance cannot be definitely resolved. However, the results clearly demonstrate that the antiapoptotic effect of patient serum on neutrophils is accompanied by an intrinsic upregulation of AAT protein. To what extend AAT expressed by neutrophils contributes to total AAT serum levels cannot be deduced from these results.

### Modulation of Mcl-1 turnover by AAT in serum-primed neutrophils

**Fig 6:**
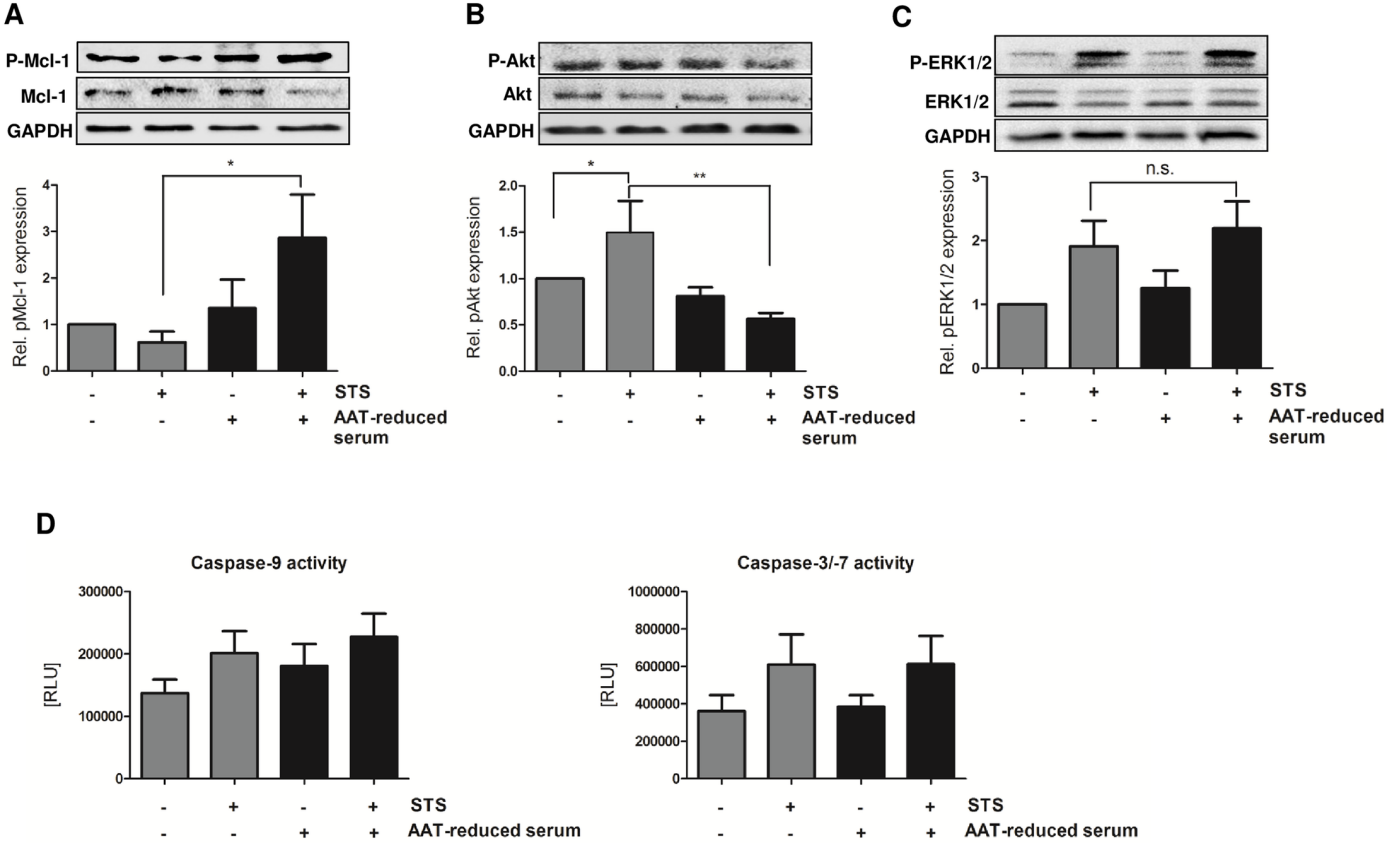
Effect of AAT on Mcl-1 phosphorylation, the activity of MAP kinases and caspases. Neutrophils from healthy volunteers (2.5 × 10^6^/ml) were cultured in medium supplemented with patient serum (3 mg protein/ml; 1%) and those containing low levels of AAT (AAT-reduce serum; 1%) in the presence of STS (0.2 μM). After 3 h, the expression of pMcl-1 (**A,** n = 7), pAkt (**B,** n = 8) and pERK1/2 (**C,** n = 10) were analyzed by western blot. Expression levels of the phosphorylated proteins were normalized to that of the unphosphorylated forms. GAPDH was used as loading control. One representative blot is displayed. *p<0.05; **p<0.01; n.s. = not significant. **D.** After 4 h of incubation the activities of caspase-9 and caspase-3/-7 were quantified. Results are presented as means ± SEM of eight independent experiments. No significant differences were found (one-way ANOVA with Newman keuls post-hoc test).

We next focused on defining how AAT impedes STS-induced intrinsic apoptosis and analyzed the effects of AAT on Mcl-1 protein stability, which is critical for neutrophil survival, as well as on the activity of Akt and ERK1/2. For this, AAT was first immunodepleted from pooled patient serum before addition to the cell culture media and STS treatment. As already displayed in Fig 4C, increase in apoptosis after depletion of AAT from serum was linked to a significant increase in Mcl-1 protein phosphorylation after 3 h of incubation (Fig 6A). Thus, diminution of native AAT provoked upregulation of phospho-Mcl-1 (pMcl-1; Fig 6A) accompanied by an increased protein turnover as well as accelerated intrinsic and spontaneous apoptosis (Fig 4C). AAT-reduced serum also diminished the activity of the survival kinase Akt in the absence as well as significantly in presence of STS (Fig 6B). However, ERK1/2 activity markedly increased in STS-treated cells and no significant changes could be observed in the absence of AAT (Fig 6C). This indicates that the antiapoptotic effects of AAT are mediated by the suppression of the Akt pathway. Recently, we demonstrated that STS-induced apoptosis in neutrophils depends on both, proteasome- and caspase-dependent Mcl-1 degradation [16]. However, although STS-triggered increase in caspase activities could be blunted in patient serum-primed neutrophils, here, we did not find any effects of native AAT on caspase activation (Fig 6D). Hence, apoptosis of neutrophils cultured in AAT-reduced serum was not accompanied by an upregulation of the activity of caspase-9 or caspase-3/-7, respectively. This surprising finding strongly indicates that the antiapoptotic action of AAT is based on the inhibition of Mcl-1 degradation by the proteasomal pathway.

## Discussion

Human plasma is widely considered to be one of the richest physiological reservoir of the human proteome, with more than 30,000 distinct proteins. Of special interest are proteins released by different cell types in response to injury, such as after major trauma, and other pathological conditions. Identification of these proteins could represent important diagnostic markers of molecular pathways that may serve as targets for therapeutic intervention. In this regard, it is well documented that human serum contains many growth factors and antiapoptotic molecules and serum starvation has already been reported to provoke apoptotic cell death in many cell types [23–25]. Serum levels of antiinflammatory factors markedly increase during acute inflammation, such as SIRS, and strongly affect the survival of immune cells, including neutrophils, sustaining the inflammatory response. PKC has gained much attention as a potential target in the development of novel anti-inflammatory agents. Several experimental studies have demonstrated diminished swelling, neutrophil influx and vascular permeability in inflammatory disease models by PKC inhibitors [26–28]. Our own studies suggest that serum from SIRS patients strongly suppresses apoptosis induced by the broad range PKC inhibitor STS in several myeloid and lymphoid cell lines including primary neutrophils [18]. Notably, neutrophil apoptosis resistance under pathological conditions was linked to detrimental effects and strong reduction in neutrophil cell death was demonstrated to be associated with a greater risk of multiple organ dysfunction in severely injured patients [29]. Thus, neutrophil-linked collateral damage, accompanied by endothelial leakage and organ dysfunction, represent common complications in critical care units. Although therapeutic use of cytokine filters in SIRS patients demonstrated first beneficial effects [30], the serum factors involved in the regulation of neutrophil apoptotic pathways are actually not fully identified. Here, we identified native human AAT (also known as Serpin A1), which represents the most abundant serpin in the circulation, as a critical mediator of neutrophil intrinsic apoptosis in SIRS patients.

The liver is widely recognized to represent the major source for AAT production. During an inflammatory response, tissue concentrations of AAT may increase as much as 11-fold as a result of local synthesis by resident or invading inflammatory cells [31]. Additionally, increased AAT secretion is mainly mediated by cytokines, such as IL-6 and TNF-α, which might induce AAT expression by different magnitude [32]. In this regard, it has already been demonstrated that human alveolar macrophages can contribute to tissue AAT levels in response to inflammatory cytokines (IL-6, IL-1 and TNF-α) and endotoxins [33]. Correspondingly, AAT serum levels were markedly elevated in our cohort of severely injured patients until at least days 10–11 after admission showing direct correlation with neutrophil apoptosis resistance to STS [20], although the source of serum AAT remained unidentified. However, in accordance with the study of Clemmensen *et al*., we demonstrate here that *in vitro* stimulation of human neutrophils with inflammatory serum factors, such as G-CSF, markedly upregulates AAT expression in these cells. AAT, which is known to be stored in granules, was additionally reported to not become constitutively secreted by the cells, but rather upon stimulation with fMLP or PMA [34]. Therefore, our *in vitro* findings showing AAT accumulation in stimulated neutrophils do not exclude the possibility that circulating as well as tissue neutrophils might represent an *in vivo* source for extracellular AAT upon inflammatory insults.

AAT was originally described as a natural occurring circulating serine protease inhibitor that primarily blocks the proteolytic activity of neutrophil elastase (22). Meanwhile, it has become evident that human AAT is a multifunctional protein with antiinflammatory, cytoprotective as well as immunoregulatory properties. Many studies reported protective effects of AAT augmentation in respect of inflammation, elastase activity, superoxide production or TNF-α secretion respectively (23–25). The antiinflammatory and tissue protective functional role of AAT has been partially related to its ability to inhibit cellular apoptosis (26). However, in general, the impact of AAT on apoptotic processes seems to be multifaceted. On the one hand, AAT might induce neutrophil apoptosis by a pathway involving endoplasmic reticulum stress and TNF-α signalling [35]. On the other hand, the antiapoptotic action of AAT was previously reported to be based on the inhibition of caspases activities, such as caspase-1 and caspase-3 [36, 37]. Here, AAT withdrawal from serum significantly increased neutrophil spontaneous apoptosis, although no increase in caspases activity could be noted. In contrast, supplementation of human plasma-derived AAT to the culture medium did not modulate neutrophil apoptotic cell death, arguing for altered biological activity of AAT in SIRS patients. Indeed, multiple isoforms of AAT are known to exist [38] and AAT synthesized by neutrophils during inflammation has been reported to be different from liver-derived AAT [34]. Importantly, although our experimental findings clearly demonstrate that neutrophils from SIRS patients as well as upon *in vitro* serum stimulation express AAT, they do not explain how this newly synthesized AAT contributes to the inhibition of apoptosis. We speculate that intrinsic AAT could be important to retain long-term resistance to apoptosis inducers in the absence of various inflammatory factors. This speculation is in line with the fact that neutrophils from trauma patients with SIRS retain resistance to the intrinsic apoptosis inducer STS when cultured in patient serum-free culture medium [18].

Inhibition of STS-triggered apoptosis by AAT has previously been shown by others. In this regard, Petrache *et al*. demonstrated that primary pulmonary endothelial cells internalize human AAT, which co-localized with and inhibited STS-induced caspase-3 activation. In cell-free systems, AAT strongly inhibited the interaction between caspase-3 and its substrate [37]. Importantly, these observations could not be confirmed by our study. As AAT immunodepletion from patients’ serum did not result in an upregulation of caspase activities after STS treatment, despite increased apoptosis, AAT does not seem to solely affect the activation of caspases. However, it should be noticed that we were not able to deplete total AAT from patients’ serum, and therefore we cannot exclude a partial inhibitory effect by serum AAT in our experiments. Additionally, other serum factors might also mediate caspase inhibition in neutrophils. Yet, our later result suggests that AAT-mediated inhibition of intrinsic apoptosis in human neutrophils involves executors other than caspases and thus also depends on caspase-independent pathways.

In this regard, Mcl-1 expression has already been demonstrated to be critical for neutrophil survival and STS resistance [18]. Therefore, we further proved whether AAT, similarly to the whole serum fraction from SIRS patients, impedes STS-triggered apoptosis by promoting Akt activation and preventing Mcl-1 degradation. Indeed, in our experiments AAT diminution in patients’ sera led to significant downregulation of phosphorylated Akt and an increase in Mcl-1 phosphorylation, suggesting a pivotal role of Akt for AAT-triggered antiapoptotic pathways in neutrophils. Rather, we found phosphorylation of ERK1/2, which is known to promote Mcl-1 protein stabilization by phosphorylation of Thr163, to not underlie regulatory changes by serum AAT. In support of our results, ERK1/2 activation has been shown to reduce Mcl-1 turnover only in cells in which Mcl-1 degradation is not dependent on GSK-3-induced phosphorylation at Ser159 and thus on the Akt/GSK3 signaling pathway [39].

On the basis of our results, we postulate that strong PKC/Akt activation in human neutrophils during acute inflammation, in part mediated by circulating AAT, markedly reduces the sensitivity of these cells to PKC inhibitors such as STS. Our hypothesis is further supported by a report, demonstrating PKC-mediated Akt activation in neutrophils [15]. In fact, in primed neutrophils proapoptotic STS seems to trigger a counteracting survival pathway accompanied by an increase in Akt activity and diminution of apoptotic pathways. The answer to the question how AAT engages this mechanism remains elusive. Our findings demonstrate that AAT downregulation might increase sensitivity of cells to proapoptotic stimuli limiting neutrophil survival in systemic inflammation and contributing to the transient antiinflammatory response. Additionally, in our view, this is the first study showing that the antiapoptotic actions of AAT embed Mcl-1 regulatory pathways. Taken into account that AAT represents a natural circulating inhibitor of neutrophil elastase and AAT deficiency has been associated with the development of chronic obstructive pulmonary disease characterized by tissue destruction [40], the advantages and disadvantages of AAT-targeted therapies need rigorous assessment. Therefore, further studies using an *in vivo* trauma model and conditional AAT knockout mice would be required to appreciate the role of AAT in respect to pathogenesis.

## Supporting information

S1 Table Identification of AAT in the B14 fraction by mass spectrometry. (PPTX)

S2 Table Identification of AAT in the Tris-eluted supernatant by mass spectrometry. (PPTX)
