# Supplementary figures and images for: Serum α-1 Antitrypsin (AAT) antagonizes intrinsic apoptosis induction in neutrophils from patients with systemic inflammatory response syndrome
Source: PLoS One. 2017 May 11;12(5):e0177450. doi: 10.1371/journal.pone.0177450 (PMC5426753; doi:10.1371/journal.pone.0177450)

## Slide 1
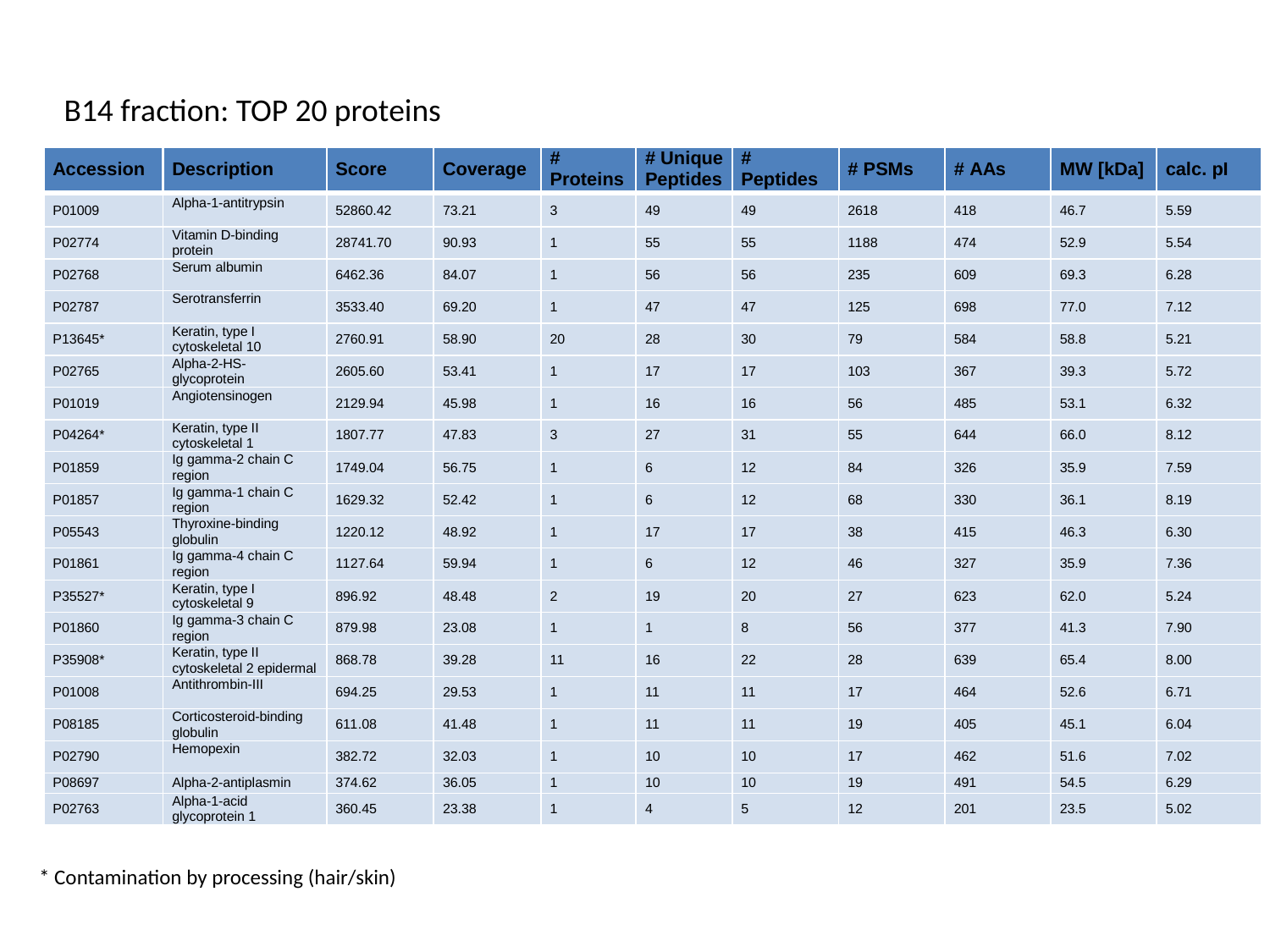

B14 fraction: TOP 20 proteins
* Contamination by processing (hair/skin)

Supplement: S1 Table — (PPTX) [file pone.0177450.s001.pptx]

## Slide 1
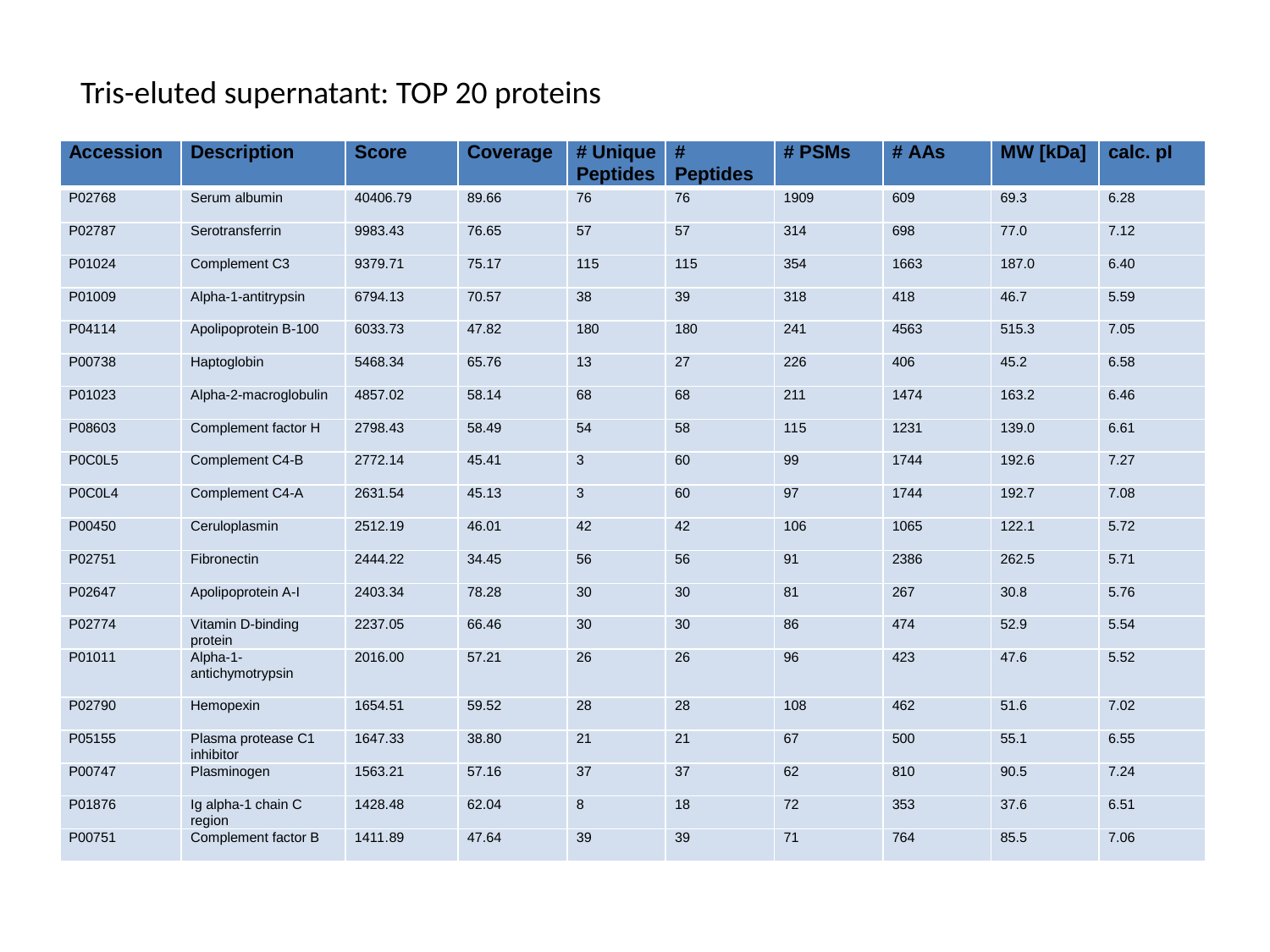

Tris-eluted supernatant: TOP 20 proteins

Supplement: S2 Table — (PPTX) [file pone.0177450.s002.pptx]
